# Detecting a potential causal relationship between plasma metabolites and myocardial infarction using bidirectional and two-step Mendelian randomization

**DOI:** 10.1038/s41598-025-04687-w

**Published:** 2025-07-02

**Authors:** Mengqi Yang, Meng Wang, Jie Li, Min Li, Xuejiao Liu, Yan Li, Yitao Xue

**Affiliations:** 1 Department of Cardiology, Weifang Hospital of Traditional Chinese Medicine, Shandong Second Medical University, Weifang, China; 2 Cardiovascular Disease Laboratory, Weifang Hospital of Traditional Chinese Medicine, Shandong Second Medical University, Weifang, China; 3 Shandong Academy of Chinese Medicine, Jinan, China; 4 Department of Gastroenterology, Shuguang Hospital, Shanghai University of Traditional Chinese Medicine, Shanghai, China; 5 Department of Cardiology, Affiliated Hospital of Shandong University of Traditional Chinese Medicine, Jinan, China

**Keywords:** Plasma metabolites, Myocardial infarction, Immune cells, Mendelian randomisation, Risk factors, Cardiovascular diseases

## Abstract

Some studies have shown that plasma metabolites may be associated with myocardial infarction (MI); however, the causal relationship between plasma metabolites and MI, as well as the potential mediating role of immune cells, remains unclear. This Mendelian randomisation (MR) study utilised large-scale genome-wide association study (GWAS) summary data encompassing 1400 plasma metabolites (n = 8299), 731 immune cell traits from the GWAS Catalog consortium (n = 3757), and MI cases and controls from the FinnGen consortium (cases: n = 26,060; controls: n = 343,079). Using bidirectional MR analysis, we assessed the causal links between plasma metabolites and MI, and between immune cells and MI, excluding reverse causality. Five MR methods were applied, with inverse variance weighting used as the primary analytical approach. In addition, we conducted two-step MR to identify potential immune cell mediators. We identified 44 positive and 33 negative causal associations between genetic liability to plasma metabolites and MI. Of these, only the association between 3β-hydroxy-5-cholestenoate (OR = 0.909; 95% CI 0.871–0.950; P = 1.84 × 10^–5^) and MI remained statistically significant after Bonferroni correction. Additionally, eight positive and five negative causal associations were observed between immune cells and MI. Among them, HLA-DR on dendritic cells (OR = 1.039; 95% CI 1.020–1.057; P = 2.84 × 10^–5^) and HLA-DR on plasmacytoid dendritic cells (OR = 1.031; 95% CI 1.016–1.047; P = 4.33 × 10^–5^) were identified as risk factors for MI after correction. Notably, bidirectional MR revealed that the glutamine conjugate of C6H10O2 (1) (OR = 1.125; 95% CI 1.042–1.215; P = 0.003) was causally associated with increased MI risk, with no evidence of reverse causality or heterogeneity. In the two-step MR analysis, positive associations were found between this metabolite and HLA-DR on CD33-HLA-DR + cells (OR = 1.302; 95% CI 1.014–1.671; P = 0.038), and between the immune trait HLA-DR on CD33-HLA-DR + (OR = 1.035; 95% CI 1.010–1.060; P = 0.005) and MI. Furthermore, mediation analysis indicated that 7.68% of the effect of the metabolite on MI was mediated through HLA-DR on CD33-HLA-DR^+^. Plasma metabolites and immune cells demonstrated causal associations with myocardial infarction. Moreover, immune cells acted as mediators in the causal pathway from plasma metabolites to myocardial infarction.

## Introduction

Acute myocardial infarction (MI) is one of the leading causes of death and disability worldwide^1^, although its prognosis has improved significantly in recent decades owing to advances in early reperfusion strategies, medical therapy, and standardised care^2–4^. Recent studies have revealed associations between plasma metabolites and MI, plasma metabolites and immune cells, and immune cells and MI. Metabolic processes can influence disease risk and may provide therapeutic targets^5^. For instance, Wang et al. found that individuals with a high plasma abundance of dU, homoserine, or methionine have an increased risk of MI^6^. Metabolism plays a dominant role in immune regulation. Hu et al. uncovered a previously uncharacterised role for creatine in macrophage polarisation by modulating cellular responses to cytokines such as IFN-γ and IL-4^7^. Monocytes and macrophages are associated with MI and exhibit a pro-inflammatory phenotype^8^. However, the relationship between plasma metabolites, immune cells, and MI remains elusive.

Mendelian randomisation (MR) is an approach that uses genetic variants as instrumental variables (IVs) to establish causal relationships between exposures and clinical outcomes, while controlling for confounding and reducing reverse causality bias^9^. Furthermore, the growing number of genome-wide association studies (GWAS) has identified numerous genotype–phenotype associations, allowing MR to be used as a powerful tool to infer causal links between plasma metabolites, immune cells, and MI. In this study, we conducted a bidirectional MR analysis and mediation MR using summary statistics from the most extensive and up-to-date GWAS datasets on plasma metabolites, immune cells, and MI. We aimed to dissect the complex associations between these variables and provide valuable insights into their causal relationships.

## Materials and methods

### Study design

In this study, we used publicly available GWAS data to investigate the genetic association between plasma metabolites and MI. We assessed the direct effects of plasma metabolites on MI, as well as the indirect effects mediated by immune cells.

**Fig. 1:**
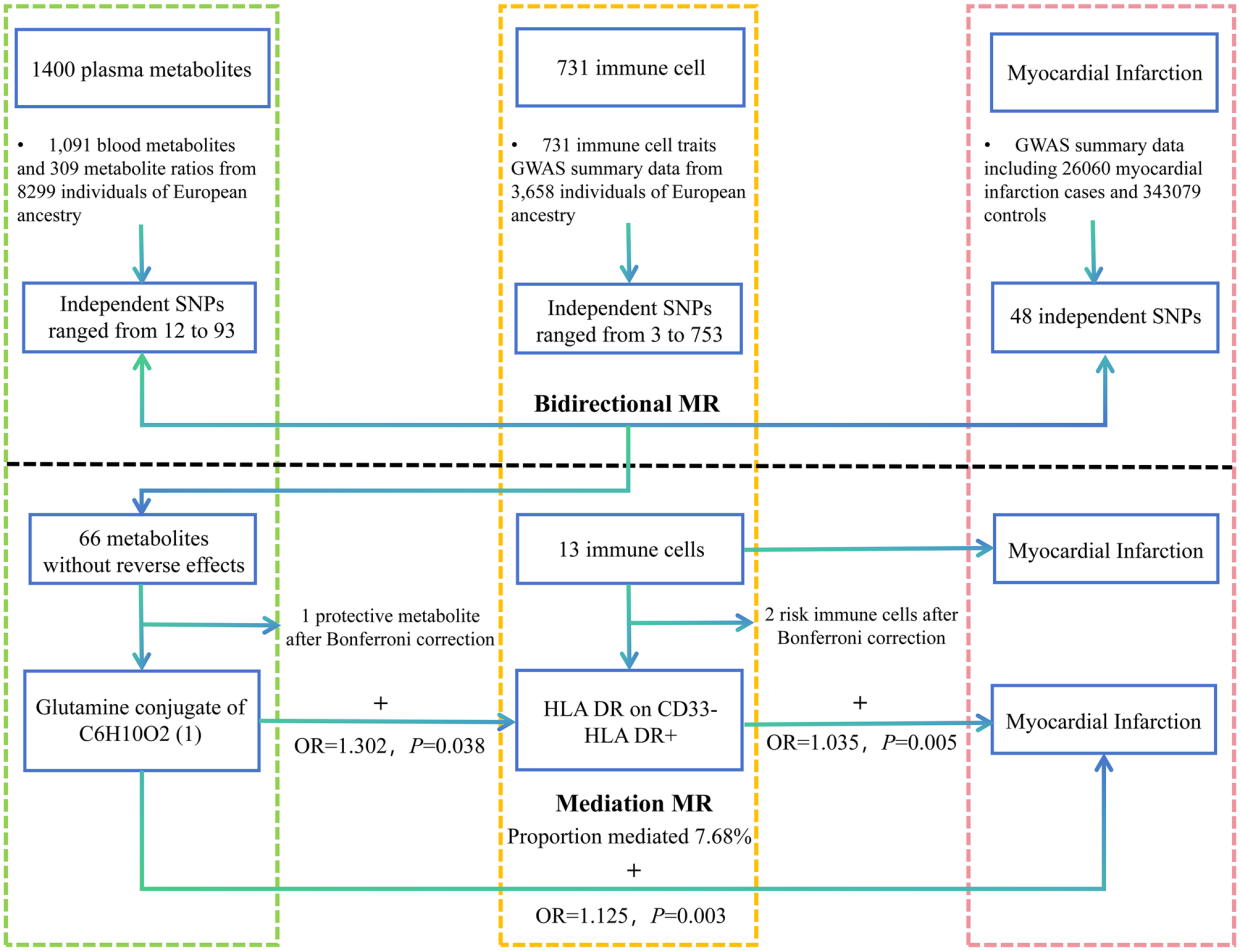
Study overview.

We conducted a two-step MR analysis to explore the potential mediating role of immune cells in the relationship between plasma metabolites and MI. The causal effect of plasma metabolites on immune cells was assessed first, followed by an evaluation of the causal effect of immune cells on MI (Fig. 1).

This study adhered to the reporting guidelines of the Strengthening the Reporting of Observational Studies in Epidemiology using Mendelian Randomisation (STROBE-MR)^9^.

### Data source

The genetic data for plasma metabolites were obtained from the latest GWAS summary statistics, in which researchers curated and analysed genome-wide association studies of 1091 blood metabolites and 309 metabolite ratios from 8299 individuals^5^. The genetic data for 731 immune cell traits were derived from the GWAS Catalog (Ebi-a-GCST90001391 to Ebi-a-GCST90002121)^10,11^, comprising seven cell panels: B cells, conventional dendritic cells (cDCs), mature T cell stages, monocytes, myeloid cells, TBNK (B cells, natural killer cells, and T cells), and T regulatory (Treg) panels. The GWAS summary data for MI were obtained from the tenth release of the FinnGen consortium (https://r10.finngen.fi/), comprising 26,060 MI cases and 343,079 controls^12^. It was a prospective cohort study in which patients were screened using the International Classification of Diseases (ICD) codes for MI. All participants in this dataset were of European ancestry.

This study is a secondary analysis of publicly available GWAS summary data. Ethical approval had been granted for each of the original GWAS datasets. Moreover, no individual-level data were used in this study; thus, no additional ethical review board approval was required.

### Instrumental variables selection and data harmonisation

We used single nucleotide polymorphisms (SNPs) as IVs to investigate the causal relationships between exposures and outcomes at the genetic level. SNPs were initially included if they demonstrated a significant association with the exposure at the genome-wide level (P < 5 × 10^–8^ for myocardial infarction [MI]; P < 1 × 10^–5^ for plasma metabolites and immune cells)^13,14^. These are commonly used thresholds in GWAS to minimise weak instrument bias while retaining a sufficient number of IVs.

Subsequently, SNPs likely to be affected by linkage disequilibrium (LD) were excluded from the MR analysis. The specific criterion for SNP selection involved applying a threshold of R^2^ < 0.001 and a window of 10,000 kb, eliminating SNPs located within a 10 Mb region that exhibited LD with the most significant SNP (R^2^ > 0.001)^15^. To assess instrument strength, we also calculated the F-statistic for each SNP using the formula: F = R^2^(N − 2)/(1 − R^2^). An *F*-statistic greater than 10 was considered indicative of adequate instrument strength, in accordance with the commonly accepted rule-of-thumb to avoid weak instrument bias in MR analysis^16^. An essential step in MR analysis is to ensure that the effects of SNPs on the exposure align with the same allele as the effects on the outcome. SNPs exhibiting palindromic or ambiguous characteristics were systematically excluded from the IVs used in our analysis. These adjustments enabled us to access a broader and more reliable set of SNPs associated with plasma metabolites, immune cells, and MI.

### MR analysis and mediation analysis

We performed a comprehensive two-sample bidirectional MR analysis to assess reciprocal causality between plasma metabolites and MI. Following this, a mediation analysis was conducted using a two-step MR approach to identify immune cells that mediate the relationship between plasma metabolites and MI.

### Two-sample MR analysis

To estimate the causal effects of plasma metabolites and immune cells on MI, we performed separate two-sample MR analyses. The inverse-variance weighted (IVW) method was used as the primary analytical approach due to its robustness in integrating effect estimates from individual SNPs into a single aggregated effect size^17^. The IVW method provides high statistical power and low bias when the instrumental variable assumptions are satisfied. To validate the robustness of the IVW-based results and ensure a comprehensive analysis, we employed MR-Egger, weighted median, simple mode, and weighted mode methods as complementary approaches. MR-Egger not only provides MR estimates but also detects directional pleiotropy through its intercept term; however, its estimation accuracy is relatively low^18,19^. The weighted median yields a consistent estimate under the assumption that at least 50% of the instrumental variables are valid^20^. Although the simple mode method is less powerful than IVW, it offers robustness in the presence of pleiotropy^21^. The weighted mode is sensitive to bandwidth selection, which can affect mode estimation accuracy^22^. MR results were reported as odds ratios (ORs) with corresponding 95% confidence intervals (CIs). Results were considered statistically significant when the *P*-value for IVW was less than 0.05 and the direction of effects for IVW and MR-Egger methods were consistent.

### Bidirectional causality analysis

To evaluate bidirectional causal effects between plasma metabolites and MI, we used MI as the exposure and plasma metabolites or immune cells associated with MI as the outcomes. SNPs significantly associated with MI (P < 5 × 10^–8^) were selected IVs.

### Mediation analysis

Plasma metabolites and immune cells with significant causal effects on MI, as identified in the two-sample MR analysis, were included in the mediation analysis. Indirect effects were calculated by multiplying β1 by β2, where β1 represents the MR effect of plasma metabolites on the mediators, and β2 denotes the MR effect of the mediators on MI^23^. This analysis provided insights into the influence of plasma metabolites on MI that is mediated by immune cells within the causal pathway.

### Sensitivity analysis

Cochran’s Q test was conducted to assess heterogeneity across SNPs. A P-value > 0.05 in this analysis indicates non-significant heterogeneity, supporting consistency across the genetic variants^24^. Leave-one-SNP-out analysis was performed to identify influential SNPs and assess the robustness of the results^19^. In addition, we applied MR-PRESSO and MR-Egger regression to evaluate potential horizontal pleiotropy. MR-PRESSO was used to identify significant outliers and to correct for horizontal pleiotropic effects by removing them^25^. MR-Egger regression was employed to detect potential directional pleiotropy and to assess its impact using the intercept test; a *P*-value > 0.05 suggests the absence of pleiotropy^19^. To correct for multiple testing, Bonferroni correction was applied^26^. A two-sided *P*-value passing the correction threshold was defined as statistically significant: 0.000036 (0.05/1,294) for plasma metabolites and 0.000068 (0.05/731) for immune cells. Associations with P-values < 0.05 were considered suggestively significant.

All analyses were conducted using R software (version 4.4.0; R Foundation for Statistical Computing, Vienna, Austria). The “TwoSampleMR” R package (version 0.6.2; Gibran Hemani, University of Bristol, UK) was used to perform the two-sample MR analyses.

## Results

### Genetic instruments for exposures

The number of SNPs serving as IVs varied across datasets. For the 1,294 plasma metabolites (106 were excluded after selection), the number of SNPs ranged from 12 to 93 (median: 24). For the 731 immune cell traits, SNP counts ranged from 3 to 753 (median: 23). In the reverse MR analysis of MI on plasma metabolites, 48 SNPs were used for MI. The median *F*-statistic was 21.20 (range: 19.50–2297.79) for plasma metabolites, 19.54 (range: 19.54–3159.29) for immune cells, and 44.48 (range: 29.73–403.41) for MI (Supplemental Tables 1–3). All IVs had *F*-statistics > 10, indicating no bias due to weak instruments. These results confirm the robustness of the selected IVs, supporting their suitability for MR investigations.

**Table 1:** MR analysis of causal effect of CA on MI.

Exposure	Method	SNP	*P-*value	OR (95% CI)	β
CA	MR Egger	27	4.28E−03	0.860 (0.780,0.925)	− 0.151
	Weighted median	27	0.001	0.905 (0.851,0.962)	− 0.100
	IVW	27	1.84E−05	0.909 (0.871,0.950)	− 0.095
	Simple mode	27	0.014	0.860 (0.769,0.962)	− 0.151
	Weighted mode	27	0.001	0.896 (0.843,0.951)	− 0.110

CA: 3β-hydroxy-5-cholestenoate; IVW: inverse-variance weighted; SNP: single nucleotide polymorphism.

### Bidirectional causal effects of MI on plasma metabolites

#### Causal effects of plasma metabolites on MI

**Fig. 2:**
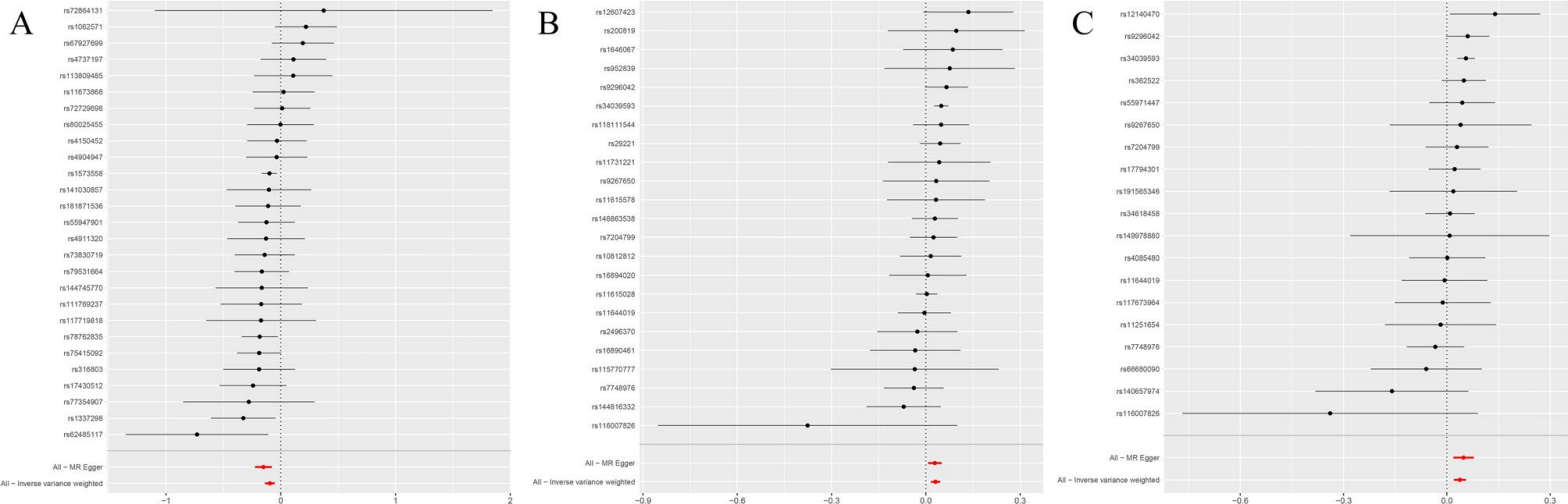
Forest plot. (**A**) MR effect size of CA on MI. (**B**) MR effect size of HLA-DR on plasmacytoid DCs on MI. (**C**) MR effect size of HLA-DR on DCs on MI.

**Fig. 3:**
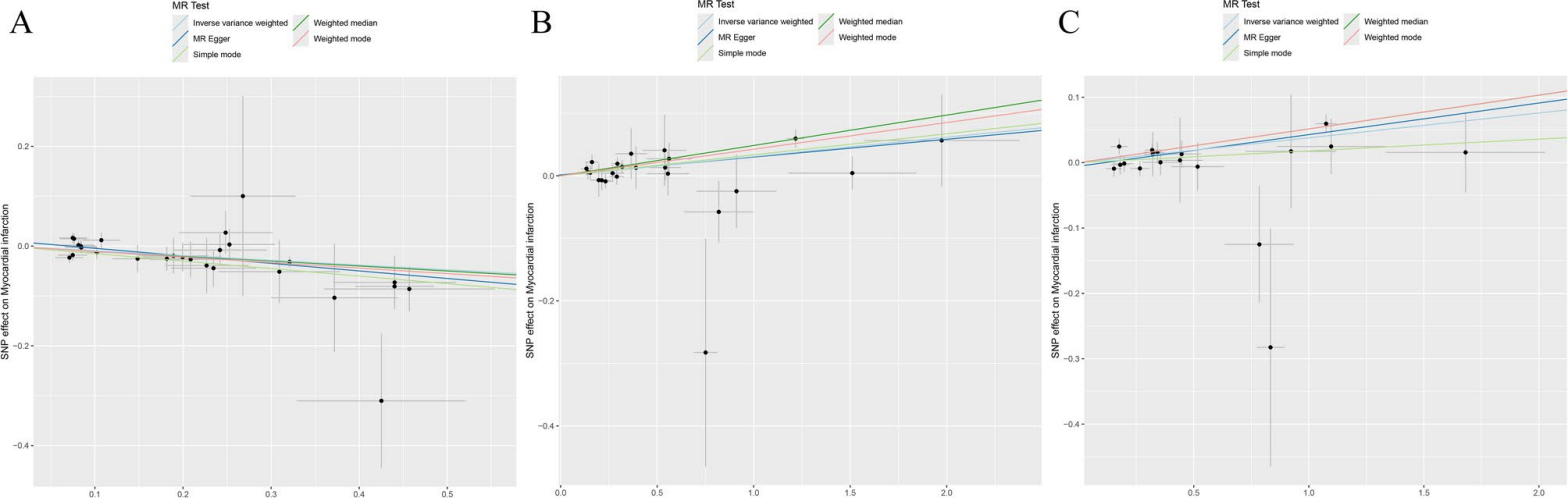
Scatter plot. (**A**) SNP effects on CA. (**B**) SNP effects on HLA-DR on plasmacytoid DCs. (**C**) SNP effects on HLA-DR on DCs.

**Fig. 4:**
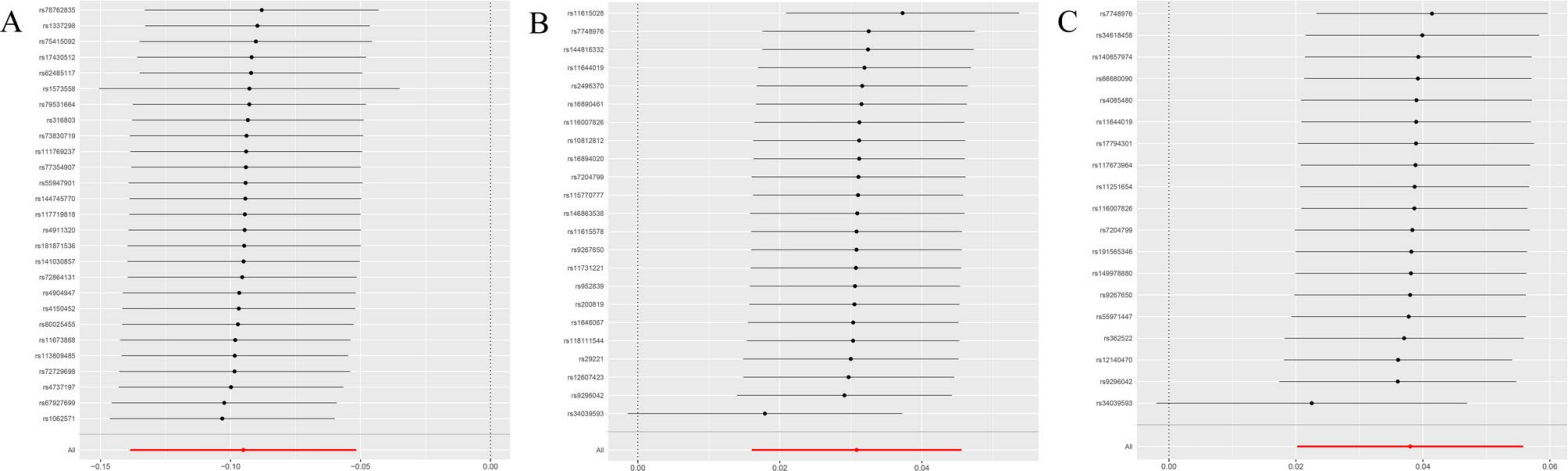
Sensitivity analysis. (**A**) MR leave-one-out sensitivity analysis of CA on MI. (**B**) MR leave-one-out sensitivity analysis of HLA-DR on plasmacytoid DCs on MI. (**C**) MR leave-one-out sensitivity analysis of HLA-DR on DCs on MI.

In our genetic analysis of plasma metabolites associated with MI, we used 27,262 SNPs as IVs. Multiple MR analysis methods indicated potential causal associations between 77 plasma metabolites and the risk of MI (Supplemental Table 4). IVW method identified 44 metabolites as risk factors for MI and 33 as protective factors. After applying Bonferroni correction for multiple comparisons, only the association between 3β-hydroxy-5-cholestenoate (CA) and MI (OR = 0.909; 95% CI 0.871–0.950; P = 1.84 × 10^–5^) remained statistically significant (Table 1; Figs. 2A, 3A, 4A).

### Causal effects of MI on plasma metabolites

When assessing the causal effects of MI on 77 plasma metabolites, we identified evidence suggesting that a genetic predisposition to MI was associated with 10 plasma metabolites (Supplemental Table 5). The most statistically significant association was observed for campesterol, with an odds ratio (OR) of 1.127 (95% confidence interval [CI]: 1.027–1.237; P = 0.012). A total of 67 plasma metabolites were associated with MI, comprising 29 positive and 38 negative effects, with no evidence of reverse causality (Supplemental Table 6).

Notably, MI was positively associated with X-21467 (OR = 1.077; 95% CI 1.011–1.148; P = 0.022), deoxycholic acid glucuronide (OR = 1.070; 95% CI 1.006–1.138; P = 0.032), the alpha-ketoglutarate to alpha-ketobutyrate ratio (OR = 1.080; 95% CI 1.015–1.149; P = 0.016), and the glycine to alanine ratio (OR = 1.072; 95% CI 1.009–1.138; P = 0.024). Each of these metabolites exhibited a protective effect against MI when considered as exposures in the reverse MR analysis. In contrast, campesterol, ethyl beta-glucopyranoside, orotidine, and the salicylate to caprylate (8:0) ratio were identified as risk factors for MI, and MI was positively associated with all of them. However, no plasma metabolites remained significantly associated with MI after applying the Bonferroni correction.

Heterogeneity testing for the associations between plasma metabolites and MI revealed that all *P*-values were > 0.05, and there was no evidence of horizontal pleiotropy. These findings enhance the reliability of the IVW results (Supplemental Table 7).

### Mediation analysis of plasma metabolites, immune cells, and MI

In this MR analysis of 731 immune cell traits associated with MI, 13 immune cells were found to be causally associated with MI (Supplemental Table 8). IVW analysis indicated that eight immune cells were risk factors for MI, whereas five were protective. After applying Bonferroni correction for multiple comparisons, only two associations remained statistically significant. IVW analysis revealed that genetically predicted HLA-DR on dendritic cells (DCs) (OR = 1.039; 95% confidence interval [CI]: 1.020–1.057; P = 2.84 × 10^–5^) and HLA-DR on plasmacytoid DCs (OR = 1.031; 95% CI 1.016–1.047; P = 4.33 × 10^–5^) were significantly associated with increased MI risk (Table 2; Figs. 2B–C, 3B–C, 4B–C).

**Table 2:** MR analysis of causal effects of immune cells on MI.

Exposure	Method	SNP	*P-*value	OR (95% CI)	β
HLA DR on DC	MR Egger	19	0.006	1.050 (1.019,1.081)	0.048
	Weighted median	19	2.14E−05	1.053 (1.028,1.078)	0.052
	IVW	19	2.84E−05	1.039 (1.020,1.057)	0.038
	Simple mode	19	0.399	1.018 (0.977,1.061)	0.018
	Weighted mode	19	0.0002	1.053 (1.030,1.077)	0.052
HLA DR on plasmacytoid DC	MR Egger	23	0.018	1.029 (1.001,1.052)	0.028
	Weighted median	23	9.40E−06	1.050 (1.027,1.072)	0.049
	IVW	23	4.33E−05	1.031 (1.016,1.047)	0.031
	Simple mode	23	0.120	1.034 (0.993,1.077)	0.034
	Weighted mode	23	0.001	1.043 (1.021,1.066)	0.043

IVW: inverse-variance weighted; SNP: single nucleotide polymorphism.

In this MR analysis, both plasma metabolites and immune cells exhibited causal effects on MI. Furthermore, immune cells appeared to play a mediating role in the causal pathway linking plasma metabolites to MI (Supplemental Table 9). Importantly, no evidence of heterogeneity or horizontal pleiotropy was detected, and no outliers were identified.

**Fig. 5:**
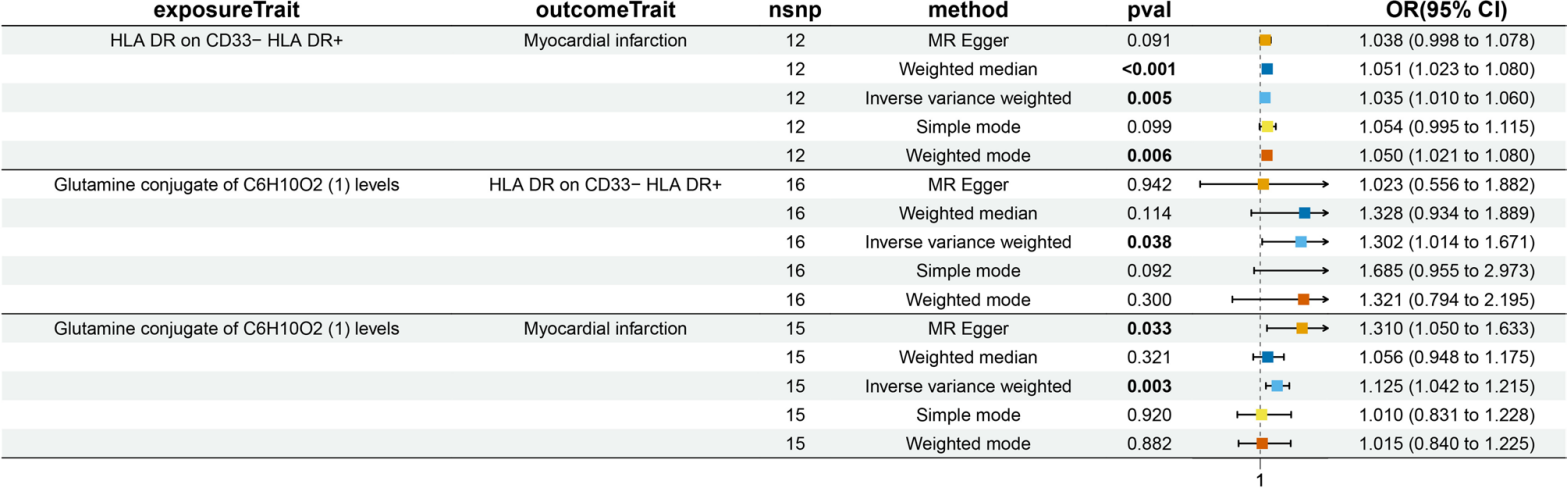
Mediation analysis.

**Fig. 6:**
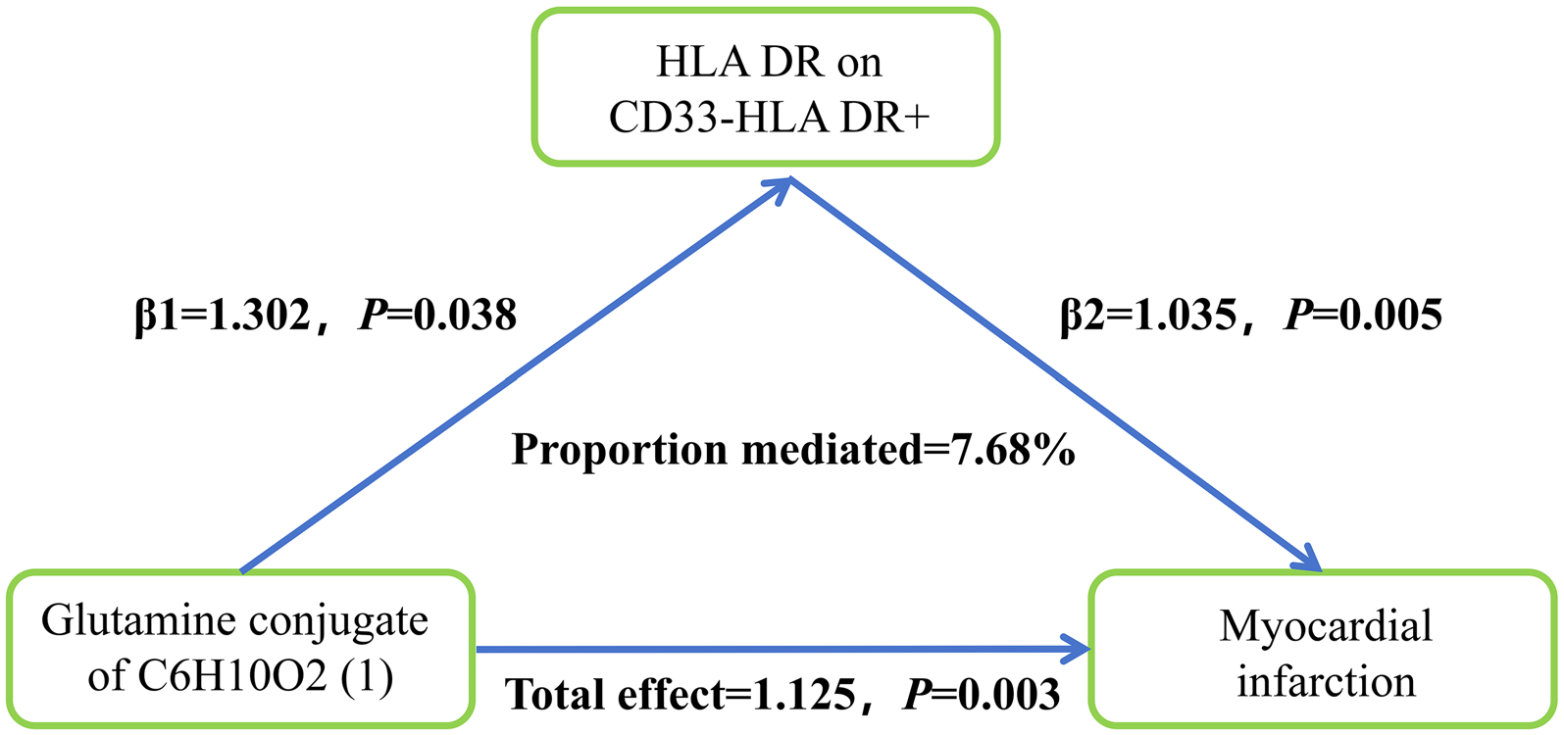
Mediation analysis. The β1 coefficient represents the effect of the metabolite on immune cells; The β2 coefficient represents the effect of immune cells on MI.

MR analysis revealed statistically significant causal relationships among the glutamine conjugate of C6H10O2 (1), myocardial infarction (MI), and HLA-DR expression on CD33-HLA-DR + cells. Specifically, the glutamine conjugate of C6H10O2 (1) exhibited a causal effect on MI mediated by HLA-DR expression on CD33-HLA-DR + cells. The β1 and β2 coefficient were estimated using the IVW method (Figs. 5, 6).

## Discussion

In this MR investigation, we identified a suggestive causal association between 77 plasma metabolites and MI, and between 13 immune cell traits and MI. Following rigorous selection criteria, we determined that one plasma metabolite and two immune cell traits demonstrated statistically significant causal effects on MI. Through advanced analytical approaches, including bidirectional MR and two-step MR, we uncovered a potential mechanistic pathway involving the immune cell trait HLA-DR on CD33-HLA-DR^+^ cells in the association between the plasma metabolite glutamine conjugate of C6H10O2 (1) and MI. We propose that this metabolite may contribute to an increased risk of MI by upregulating the expression of HLA-DR on CD33-HLA-DR + cells. To our knowledge, this study is the first to comprehensively investigate the genetic associations between plasma metabolites and MI, revealing the critical role of metabolism in cardiovascular risk. These findings may offer novel perspectives for the prevention and treatment of MI, particularly through interventions targeting metabolic and immune pathways.

Plasma metabolic dysregulation plays a crucial role in the pathophysiology of MI. Accumulating evidence indicates that plasma metabolites can influence multiple aspects of disease pathogenesis in patients with ischaemic heart disease and may accelerate disease progression. In particular, previous studies have demonstrated that plasma metabolites affect cardiomyocyte apoptosis^27^, vascular endothelial function^28^, organelle substructure integrity^29^, and abnormal energy metabolism^30^. In human studies, elevated plasma levels of 15-hydroxyeicosatetraenoic acid and 3-hydroxybutyric acid, along with reduced levels of lysophosphatidylcholine (16:0), ceramide (d18:0/18:0), and tryptophan, have been reported in patients with QRS fragmentation—suggesting a pathogenetic role for plasma metabolites in cardiac electrical remodelling^31^. However, until now, the causal relationship between plasma metabolites and MI had not been clearly established.

Our findings implicate CA as a protective factor, and glutamine conjugate of C6H10O2 (1) as a risk factor for MI. CA has previously been reported as an important biomarker of several severe diseases. It functions as an endogenous epigenetic regulator, modulating lipid metabolism through epigenomic modifications in human hepatocytes^32^. In contrast, the glutamine conjugate of C6H10O2 (1) has been associated with obesity-related neuropathy^33^. No previous studies have reported associations between CA or glutamine conjugate of C6H10O2 (1) and MI; thus, these findings warrant further investigation. Our reverse MR analysis identified that MI may causally increase the levels of campesterol, ethyl beta-glucopyranoside, orotidine, and the salicylate to caprylate (8:0) ratio, suggesting reverse causal effects between these metabolites and MI. The serum level of campesterol has been used to assess cholesterol absorption^34^, while orotidine has been identified as a novel biomarker of increased cardiovascular disease (CVD) risk in patients with type 2 diabetes^35^. Furthermore, our MR analysis also determined associations between ethyl beta-glucopyranoside and salicylate to caprylate (8:0) ratio and increased risk of MI, although the specific contributions of these metabolites to MI pathogenesis remain to be elucidated. Although plasma metabolites have previously been associated with MI^6^, the metabolites identified in this MR study have not been reported in earlier investigations^36^.

Our MR study presents compelling genetic evidence implicating 13 immune cell traits in the aetiology of MI. Among these, HLA-DR on DCs and HLA-DR on plasmacytoid DCs exhibited significant causal effects on MI—a finding that differs from the study conducted by Kretzschmar D et al.^37^. Their investigation showed that circulating dendritic cell precursors were significantly reduced in patients with acute myocardial infarction (AMI), with a more pronounced reduction observed in patients with ST-segment elevation myocardial infarction. This was accompanied by a marked increase in serum inflammatory cytokines in AMI patients, suggesting potential adaptive conversion of DCs or their recruitment to sites of injury^38^. There are various potential confounders and biases inherent in bidirectional causality analyses, including overlapping genetic variants, horizontal pleiotropy, and unidentified factors. However, sensitivity analyses—such as heterogeneity testing, MR-Egger regression, and MR-PRESSO—can mitigate the influence of such confounding on causal inference.

Our findings also support the hypothesis that HLA-DR on CD33-HLA-DR + cells acts as a mediator in the causal pathway linking glutamine conjugate of C6H10O2 (1) to MI. CD33 is a myeloid differentiation antigen, expressed by early myeloid progenitors and retained during myelomonocytic differentiation^39^. In humans, myeloid-derived suppressor cells (MDSCs) are commonly characterised by CD11b^+^CD33^+^HLA-DR^low expression, reflecting their immunosuppressive phenotype^40^. MDSCs have demonstrated potent immunosuppressive effects in the context of AMI, suggesting that CD33-HLA-DR + cells may instead promote an inflammatory response. Our MR analysis identified an association between HLA-DR on CD33-HLA-DR + cells and increased MI risk. Nevertheless, the precise functional role and pathological mechanisms involving this immune trait in MI pathogenesis warrant further investigation.

Our mediation analyses provide genetic evidence linking plasma metabolites with immune cell traits. To our knowledge, this study is the first to establish a direct causal connection between the glutamine conjugate of C6H10O2 (1) and HLA-DR on CD33-HLA-DR + cells. Although prior research has suggested general associations between plasma metabolites and immune cells, none have specifically linked the glutamine conjugate of C6H10O2 (1) with this immune trait. Hu et al. reported that correlation analysis of serum metabolites with cytokines and chemokines revealed strong associations. For example, arginine was strongly associated with CRS-associated inflammatory cytokines, including IL-6, IL-1β, M-CSF, IL-12p70, and IFN-α2. It was therefore hypothesised that key metabolic pathways may contribute to the regulation of pro-inflammatory cytokine and chemokine secretion^41^. Together with our findings, this study helps elucidate the complex causal interplay between plasma metabolites, immune cell traits, and the pathogenesis of inflammatory diseases. Plasma metabolites and immune cell traits causally associated with MI represent promising therapeutic targets and underscore the need for further translational research.

Our MR study, which explores the relationship between plasma metabolites and MI, is subject to several limitations. Primarily, the generalisability of our findings is constrained by the exclusive inclusion of participants of European ancestry, which may not accurately represent causal relationships between plasma metabolites and immune cells in more diverse populations. In addition, the associations identified between specific metabolites and MI risk are preliminary and should be interpreted with caution. The MR methodology employed may not fully capture complex or nonlinear interactions between plasma metabolites and MI. Furthermore, the currently available GWAS sample sizes for MI are limited, which may inadvertently increase the likelihood of false-negative results. Future research using larger and more diverse GWAS datasets is essential to clarify the complex role of plasma metabolites in MI pathogenesis. Different sensitivity analysis methods also possess inherent limitations. The relatively low statistical power of the MR-Egger method for causal estimation means that it requires larger sample sizes to reliably detect significant effects. The weighted median method, while robust to certain violations of instrumental variable assumptions, necessitates sorting and weighting the effect sizes of all SNPs, which may increase the computational burden in practical applications. Nevertheless, the use of multiple complementary MR methods and sensitivity analyses allows for better mitigation of pleiotropy and other biases, enhancing the accuracy and reliability of causal inferences. Lastly, the results of the mediation analysis did not withstand the stringent thresholds imposed by Bonferroni correction, despite the genetically supported causal relationships. However, the hypothesis-driven nature of MR enables the detection of biologically relevant causal associations that may not always meet strict multiple-testing criteria^42^. Our exploratory findings provide novel genetic evidence supporting the immunometabolic basis of MI and underscore the need for continued investigation in this area.

## Conclusions

This study evaluated the causal relationships between plasma metabolites, immune cell traits, and MI. We identified a significant role for both plasma metabolites and immune cells in the pathogenesis of MI. These findings underscore the importance of further investigating the mechanisms underlying the interaction between metabolic and immune pathways in MI. Moreover, our results provide novel insights that may inform the development of immune cell-targeted therapeutic strategies for the prevention and treatment of MI.

## Supplementary Information

Supplementary Table 1.

Supplementary Table 2.

Supplementary Table 3.

Supplementary Table 4.

Supplementary Table 5.

Supplementary Table 6.

Supplementary Table 7.

Supplementary Table 8.

Supplementary Table 9.

## Data Availability

All data used in the present study were obtained from genome-wide association study summary statistics which were publicly released by genetic consortia. Data for this study are available upon reasonable request by contacting the corresponding author.

## References

[1] Ramachandra, C. J. A., Hernandez-Resendiz, S., Crespo-Avilan, G. E., Lin, Y. H. & Hausenloy, D. J. Mitochondria in acute myocardial infarction and cardioprotection. *EBioMedicine* **57**, 102884 (2020). 32653860 10.1016/j.ebiom.2020.102884 PMC7355051

[2] Ozaki, Y. et al. CVIT expert consensus document on primary percutaneous coronary intervention (PCI) for acute myocardial infarction (AMI) update 2022. *Cardiovasc. Interv. Ther.* **37**(1), 1–34 (2022). 35018605 10.1007/s12928-021-00829-9 PMC8789715

[3] Mefford, M. T. et al. Sex-specific trends in acute myocardial infarction within an integrated healthcare network, 2000 through 2014. *Circulation* **141**(7), 509–519 (2020). 32065770 10.1161/CIRCULATIONAHA.119.044738

[4] Wang, Y., Leifheit, E. C. & Krumholz, H. M. Krumholz trends in 10-year outcomes among medicare beneficiaries who survived an acute myocardial infarction. *JAMA Cardiol.* **7**(6), 613–622 (2022). 35507330 10.1001/jamacardio.2022.0662 PMC9069341

[5] Chen, Y. et al. Genomic atlas of the plasma metabolome prioritizes metabolites implicated in human diseases. *Nat. Genet.* **55**(1), 44–53 (2023). 36635386 10.1038/s41588-022-01270-1 PMC7614162

[6] Aa, N. et al. Plasma metabolites alert patients with chest pain to occurrence of myocardial infarction. *Front. Cardiovasc. Med.* **8**, 652746 (2021). 33969016 10.3389/fcvm.2021.652746 PMC8103546

[7] Ji, L. et al. Slc6a8-mediated creatine uptake and accumulation reprogram macrophage polarization via regulating cytokine responses. *Immunity* **51**(2), 272-284.e7 (2019). 31399282 10.1016/j.immuni.2019.06.007

[8] Kologrivova, I., Shtatolkina, M., Suslova, T. & Ryabov, V. Cells of the immune system in cardiac remodeling: Main players in resolution of inflammation and repair after myocardial infarction. *Front. Immunol.* **12**, 664457 (2021). 33868315 10.3389/fimmu.2021.664457 PMC8050340

[9] Skrivankova, V. W. et al. Strengthening the reporting of observational studies in epidemiology using Mendelian randomization: The STROBE-MR statement. *JAMA* **326**(16), 1614–1621 (2021). 34698778 10.1001/jama.2021.18236

[10] Matthew, L. et al. The variant call format provides efficient and robust storage of GWAS summary statistics. *Genome Biol.*10.1101/2020.05.29.115824 (2020). 10.1186/s13059-020-02248-0 PMC7805039 33441155

[11] Orrù, V. et al. Complex genetic signatures in immune cells underlie autoimmunity and inform therapy. *Nat. Genet.* **52**(10), 1036–1045 (2020). 32929287 10.1038/s41588-020-0684-4 PMC8517961

[12] Kurki, M. I. et al. FinnGen provides genetic insights from a well-phenotyped isolated population. *Nature* **613**(7944), 508–518 (2023). 36653562 10.1038/s41586-022-05473-8 PMC9849126

[13] He, J., Huang, M., Li, N., Zha, L. & Yuan, J. Genetic association and potential mediators between sarcopenia and coronary heart disease: A bidirectional two-sample, two-step Mendelian randomization study. *Nutrients* **15**(13), 3013 (2023). 37447340 10.3390/nu15133013 PMC10346809

[14] Sanna, S. et al. Causal relationships among the gut microbiome, short-chain fatty acids and metabolic diseases. *Nat. Genet.* **51**(4), 600–605 (2019). 30778224 10.1038/s41588-019-0350-x PMC6441384

[15] Myers, T. A., Chanock, S. J. & Machiela, M. J. LDlinkR: An R package for rapidly calculating linkage disequilibrium statistics in diverse populations. *Front. Genet.* **11**, 157 (2020). 32180801 10.3389/fgene.2020.00157 PMC7059597

[16] Burgess, S., Thompson, S. G., CRP CHD Genetics Collaboration. Avoiding bias from weak instruments in Mendelian randomization studies. *Int. J. Epidemiol.* **40**(3), 755–764 (2011). 21414999 10.1093/ije/dyr036

[17] Burgess, S., Butterworth, A. & Thompson, S. G. Mendelian randomization analysis with multiple genetic variants using summarized data. *Genet. Epidemiol.* **37**(7), 658–665 (2013). 24114802 10.1002/gepi.21758 PMC4377079

[18] Bowden, J., Davey Smith, G. & Burgess, S. Mendelian randomization with invalid instruments: Effect estimation and bias detection through egger regression. *Int. J. Epidemiol.* **44**(2), 512–525 (2015). 26050253 10.1093/ije/dyv080 PMC4469799

[19] Burgess, S. & Thompson, S. G. Interpreting findings from Mendelian randomization using the MR-Egger method. *Eur. J. Epidemiol.* **32**(5), 377–389 (2017). 28527048 10.1007/s10654-017-0255-x PMC5506233

[20] Bowden, J., Davey Smith, G., Haycock, P. C., Burgess, S. & Burgess, S. Consistent estimation in Mendelian randomization with some invalid instruments using a weighted median estimator. *Genet. Epidemiol.* **40**, 304–314 (2016). 27061298 10.1002/gepi.21965 PMC4849733

[21] Milne, R. L. et al. Identification of ten variants associated with risk of estrogen-receptor-negative breast cancer. *Nat. Genet.* **49**, 1767–1778 (2017). 29058716 10.1038/ng.3785 PMC5808456

[22] Hartwig, F. P., Davey Smith, G. & Bowden, J. Robust inference in summary data Mendelian randomization via the zero modal pleiotropy assumption. *Int. J. Epidemiol.* **46**, 1985–1998 (2017). 29040600 10.1093/ije/dyx102 PMC5837715

[23] Carter, A. R. et al. Mendelian randomisation for mediation analysis: current methods and challenges for implementation. *Eur. J. Epidemiol.* **36**(5), 465–478 (2021). 33961203 10.1007/s10654-021-00757-1 PMC8159796

[24] Cohen, J. F. Cochran’s Q test was useful to assess heterogeneity in likelihood ratios in studies of diagnostic accuracy. *J. Clin. Epidemiol.* **68**(3), 299–306 (2015). 25441698 10.1016/j.jclinepi.2014.09.005

[25] Verbanck, M., Chen, C. Y., Neale, B. & Do, R. Detection of widespread horizontal pleiotropy in causal relationships inferred from Mendelian randomization between complex traits and diseases. *Nat. Genet.* **50**(5), 693–698 (2018). 29686387 10.1038/s41588-018-0099-7 PMC6083837

[26] Noble, W. S. How does multiple testing correction work?. *Nat. Biotechnol.* **27**(12), 1135–1137 (2009). 20010596 10.1038/nbt1209-1135 PMC2907892

[27] Lee, R. et al. Regulated inositol-requiring protein 1-dependent decay as a mechanism of corin RNA and protein deficiency in advanced human systolic heart failure. *J. Am. Heart Assoc.* **3**(6), e001104 (2014). 25516437 10.1161/JAHA.114.001104 PMC4338699

[28] Salmani, M. et al. Effect of l-arginine on cardiac reverse remodeling and quality of life in patients with heart failure. *Clin. Nutr.* **40**(5), 3037–3044 (2021). 33610421 10.1016/j.clnu.2021.01.044

[29] Wang, R., Li, B., Lam, S. M. & Shui, G. Integration of lipidomics and metabolomics for in-depth understanding of cellular mechanism and disease progression. *J. Genet. Genomics* **47**(2), 69–83 (2020). 32178981 10.1016/j.jgg.2019.11.009

[30] Lopaschuk, G. D., Karwi, Q. G., Tian, R., Wende, A. R. & Abel, E. D. Cardiac energy metabolism in heart failure. *Circ. Res.* **128**(10), 1487–1513 (2021). 33983836 10.1161/CIRCRESAHA.121.318241 PMC8136750

[31] Duan, W. T., Lu, Y. Q., Ma, X., Wang, L. & Qin, S. B. Application of fragmented QRS combined with plasma differential metabolites in prognosis of acute myocardial infarction. *J. Clin. Cardiol.* **37**(4), 322–327 (2021).

[32] Wang, Y. et al. Cholestenoic acid as endogenous epigenetic regulator decreases hepatocyte lipid accumulation in vitro and in vivo. *Am. J. Physiol. Gastrointest. Liver Physiol.* **326**(2), G147–G162 (2024). 37961761 10.1152/ajpgi.00184.2023 PMC11208024

[33] This data is available at the NIH Common Fund’s National Metabolomics Data Repository (NMDR) website, the Metabolomics Workbench, https://www.metabolomicsworkbench.org where it has been assigned Project ID PR000968. The data can be accessed directly via it’s Project 10.21228/M8K688. This work is supported by Metabolomics Workbench/National Metabolomics Data Repository (NMDR) (grant# U2C-DK119886), Common Fund Data Ecosystem (CFDE) (grant# 3OT2OD030544) and Metabolomics Consortium Coordinating Center (M3C) (grant# 1U2C-DK119889

[34] Matsumura, T. et al. Relationship between diabetes mellitus and serum lathosterol and campesterol levels: The CACHE study DM analysis. *J. Atheroscler. Thromb.* **30**(7), 735–753 (2023). 36171088 10.5551/jat.63725 PMC10322739

[35] Shah, H. S. et al. Serum orotidine: A novel biomarker of increased CVD risk in type 2 diabetes discovered through metabolomics studies. *Diabetes Care* **45**(8), 1882–1892 (2022). 35696261 10.2337/dc21-1789 PMC9346986

[36] Aa, N. et al. Plasma metabolites alert patients with chest pain to occurrence of myocardial infarction. *Front. Cardiovasc. Med.* **23**(8), 652746 (2021). 10.3389/fcvm.2021.652746 PMC8103546 33969016

[37] Kretzschmar, D. et al. Recruitment of circulating dendritic cell precursors into the infarcted myocardium and pro-inflammatory response in acute myocardial infarction. *Clin. Sci.* **123**, 387–398 (2012). 10.1042/CS20110561 22494099

[38] Van Vré, E. A., Van Brussel, I., Bosmans, J. M., Vrints, C. J. & Bult, H. Dendritic cells in human atherosclerosis: from circulation to atherosclerotic plaques. *Mediators Inflamm.* **2011**, 941396 (2011). 21976788 10.1155/2011/941396 PMC3184502

[39] Kim, C. H. CD33 and CD33-related siglecs in pathogen recognition and endocytosis of DC in the innate immune system. In *Glycobiology of Innate Immunology*. 10.1007/978-981-16-9081-5_12 (Springer, 2022).

[40] Zhang, M., Shi, X., Zhao, J., Guo, W. & Zhou, J. Recruitment of myeloid-derived suppressor cells and regulatory T-cells is associated with the occurrence of acute myocardial infarction. *Biomed. Rep.* **19**(2), 55 (2023). 37560314 10.3892/br.2023.1637 PMC10407468

[41] Xiao, N. et al. Integrated cytokine and metabolite analysis reveals immunometabolic reprogramming in COVID-19 patients with therapeutic implications. *Nat. Commun.* **12**(1), 1618 (2021). 33712622 10.1038/s41467-021-21907-9 PMC7955129

[42] Jiang, P. et al. Dissecting causal links between gut microbiota, inflammatory cytokines, and DLBCL: A Mendelian randomization study. *Blood Adv.* **8**(9), 2268–2278 (2024). 38507680 10.1182/bloodadvances.2023012246 PMC11117010

